# Soluble Toll-like receptor 4 is a potential serum biomarker in non-small cell lung cancer

**DOI:** 10.18632/oncotarget.9496

**Published:** 2016-05-20

**Authors:** Feng Wei, Fan Yang, Jing Li, Yu Zheng, Wenwen Yu, Lili Yang, Xiubao Ren

**Affiliations:** ^1^ Department of Immunology, Tianjin Medical University Cancer Institute and Hospital, Tianjin, PR China; ^2^ Department of Biotherapy, Tianjin Medical University Cancer Institute and Hospital, Tianjin, PR China; ^3^ National Clinical Research Center for Cancer, Tianjin, PR China; ^4^ Tianjin Key Laboratory of Cancer Immunology and Biotherapy, Tianjin, PR China

**Keywords:** soluble Toll-like receptor 4 (sTLR4), non-small cell lung cancer (NSCLC), high-mobility group box 1 (HMGB1), biomarker

## Abstract

This study investigated the clinical significance of serum soluble Toll-like receptor 4 (sTLR4) in non-small cell lung cancer (NSCLC). A total of 54 NSCLC patients and 13 healthy volunteers were enrolled from January 2012 to December 2013. The patients with NSCLC were characterized by significantly higher serum levels of sTLR4 compared with those in healthy controls (*P* < 0.01). A positive correlation between serum sTLR4 and tumor stage was found in patients with stages I–III NSCLC. However, serum sTLR4 in patients with metastatic NSCLC was significantly decreased compared with those with stage III NSCLC (*P* < 0.05). Furthermore, low serum sTLR4 was identified as a prognostic marker for poor survival of early-stage NSCLC patients who received surgical resection. In conclusion, our present study identified sTLR4 as a potential serum biomarker of NSCLC.

## INTRODUCTION

Lung cancer is one of the most frequently diagnosed cancers and the leading cause of cancer death worldwide with high metastasis and recurrence rates [1, 2]. About 87% of lung cancer is classified as non-small cell lung cancer (NSCLC), and the overall five-year survival rate for NSCLC is only 18.2%. More than half of NSCLC patients are diagnosed at an advanced stage, which is primarily due to asymptomatic presentation and lack of reliable biomarkers for early detection [3]. Therefore, sensitive biomarkers for the diagnosis and prognosis of NSCLC, as well as effectively discriminating advanced-stage disease from early-stage disease, can be of great clinical significance.

Toll-like receptors (TLRs) have recently emerged as key immunomodulators of the immune response in carcinogenesis and tumor progression. TLR4 is the first human Toll homologue to be identified and has been demonstrated to be expressed not only on immune cells but also on various cancer cells [4–10]. Significantly increased TLR4 expression has been observed in NSCLC and correlated with malignancy of cancer cells [5, 6].

Soluble forms of TLRs have been considered as negative regulators of TLR function [11]. The involvement of soluble TLR4 (sTLR4) in NSCLC has not been fully elucidated. Notably, high-mobility group nucleosome-binding protein 1 (HMGN1) and HMG box 1 (HMGB1), two major endogenous ligands of TLR4, have been identified as biomarkers of NSCLC [12–14], indicating that serum sTLR4 may also be of clinical significance in NSCLC. Lan et al. recently reported that serum sTLR4 before radiotherapy may be a potential biomarker of radiation-induced pneumonia in patients with local advanced NSCLC [15]. However, the correlation between serum sTLR4 and NSCLC progression, as well as the effects of serum sTLR4 on survival of NSCLC patients, has not been fully assessed.

In the present work, we measured the presence of sTLR4 in serum of patients with different stages of NSCLC and evaluated its possible association with clinicopathological characteristics of NSCLC patients. The prognostic value of sTLR4 was also investigated.

## RESULTS

### Serum levels of sTLR4 and HMGB1 in NSCLC patients and healthy controls

**Figure 1:**
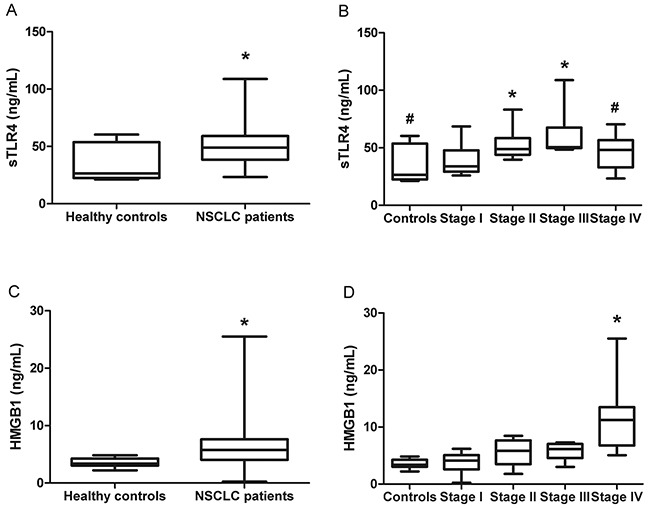
Serum levels of sTLR4 and HMGB1 in healthy controls and NSCLC patients **A.** The levels of serum sTLR4 in healthy controls and NSCLC patients. * indicates *P* < 0.05 compared with healthy controls. **B.** The levels of serum sTLR4 in healthy controls and patients with NSCLC at different TNM stages. * indicates *P* < 0.05 compared with stage I, and # indicates *P* < 0.05 compared with stage III. **C.** The levels of serum HMGB1 in healthy controls and NSCLC patients. * indicates *P* < 0.05 compared with healthy controls. **D.** The levels of serum HNGB1 in healthy controls and patients with NSCLC at different TNM stages. * indicates *P* < 0.05 compared with any other stage or controls.

The levels of sTLR4 in serum were measured by ELISA from 54 NSCLC patients and 13 healthy controls. The mean level of serum sTLR4 was significantly higher in NSCLC patients compared with that in healthy controls (49.9874±16.8638 ng/mL vs. 35.4786±15.8397 ng/mL, *P* < 0.01) (Figure 1A).

To investigate whether sTLR4 is associated with different stages of NSCLC patients, these NSCLC patients were clinically staged according to the international staging system (TNM stage), and the levels of serum sTLR4 were compared between stages. The mean levels of serum sTLR4 were 39.9433±14.7845, 52.1485±11.6008, 62.1265±19.9893, and 46.3377±13.2916 ng/mL in NSCLC patients with TNM stages I, II, III, and IV, respectively (Figure 1B). Significant differences were noted among the four stages (*P* < 0.05). The levels of serum sTLR4 were significantly higher in TNM stage II or III than in those in TNM stage I (*P* < 0.05). Serum sTLR4 level in patients with stage IV NSCLC was significantly decreased than those with stage III NSCLC (*P* < 0.05). No statistical difference in serum sTLR4 was found between TNM stages I and IV. These data demonstrated that the levels of serum sTLR4 increased as the TNM stage increased in local NSCLC and then significantly declined in patients with metastatic NSCLC.

We also accessed the serum levels of HMGB1 in the same group of NSCLC patients. Serum HMGB1 levels in NSCLC patients were significantly increased compared with those in healthy controls (6.6451±4.8100 ng/mL vs. 3.5210±0.7788 ng/mL, *P* < 0.05) (Figure 1C). Further analysis revealed that this difference mainly resulted from a dramatic increase in serum HMGB1 in stage IV NSCLC patients (Figure 1D).

### Serum sTLR4 level is associated with pathological types of NSCLC

The main clinical and pathologic characteristics of all patients are summarized in Table 1. The mean levels of serum sTLR4 in squamous cell lung carcinoma or lung adenocarcinoma patients were 56.8432±21.1424 or 46.1347±11.6985 ng/mL, respectively. Both levels were significantly higher than those in healthy controls (*P* < 0.05). Furthermore, compared with lung adenocarcinoma patients, squamous cell lung carcinoma patients also had significantly higher serum sTLR4 levels (*P* < 0.05) (Figure 2). No significant relationship was found between serum sTLR4 level and characteristics such as gender, age, or smoking status. No statistical correlation was found between sTLR4 and HMGB1 (*P* = 0.737). No significant relationship was also noted between serum HMGB1 level and pathological type, gender, age, or smoking status (data not shown).

**Table 1:** Baseline characteristics of enrolled NSCLC patients

Variables	N (%)	sTLR4 (ng/ml) Mean±SD	*P*-value
Age			
>60	27 (50)	51.4804±20.1651	0.727
≤ 60	27 (50)	53.3463±18.8476	
Gender			
Male	34 (63)	51.4067±19.5683	0.360
Female	20 (37)	47.5747±10.8852	
Smoking status			
Non-smoker	17 (31)	49.2371±10.0973	0.786
Smoker	37 (69)	50.3322±19.3127	
Pathological type^#^			
Adenocarcinoma	32 (59)	46.1347±11.6985	0.043
Squamous cell carcinoma	21 (39)	56.8432±21.1424	
Tumor stage			
Stage I	14 (26)	39.9433±14.7845	0.003
Stage II	14 (26)	52.1485±13.3620	
Stage III	13 (24)	61.5111±21.2156	
Stage IV	13 (24)	57.0300±21.9099	
Distance metastasis			
Non-metastasis	41 (76)	51.1446±17.8357	0.376
Metastasis	13 (24)	46.3377±13.2916	

# Another one case was malignant fibrous histiocytoma.

**Figure 2:**
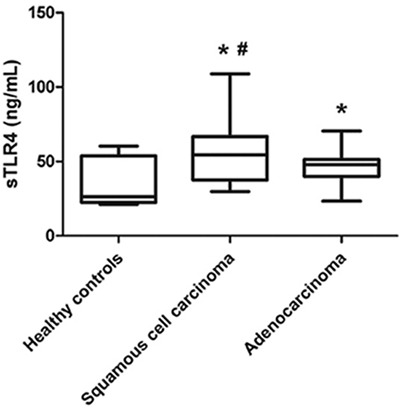
Serum levels of sTLR4 in healthy controls and NSCLC patients with different pathological types The mean levels of serum sTLR4 in either squamous cell lung carcinoma or lung adenocarcinoma patients were significantly higher than those in healthy controls. Furthermore, the mean levels of serum sTLR4 in squamous cell lung carcinoma were also significantly higher than those in lung adenocarcinoma patients. * indicates *P* < 0.05 compared with healthy controls, and # indicates *P* < 0.05 compared with lung adenocarcinoma.

### Serum sTLR4 overexpression indicates a favorable prognosis in patients with early-stage NSCLC

All the 28 NSCLC patients with TNM stage I or II received surgical resection. The association between serum sTLR4 level and clinicopathologic characteristics of these early-stage NSCLC patients is summarized in Table 2.

**Table 2:** Association between serum sTLR4 level and clinicopathologic characteristics in 28 early-stage NSCLC patients who received pneumonectomy

Variables	Early-stage patients (n=28)	sTLR4-low (n=14)	sTLR4-High (n=14)	*P*-value
Age (mean±SD)	60.4±7.5	60.2±9.5	60.5±5.0	0.922
>60	15	8	7	0.705
≤ 60	13	6	7	
Gender				
Male	17	7	10	0.246
Female	11	7	4	
Smoking status				
Non-smoker	9	5	4	1.000
Smoker	19	9	10	
Pathological type				
Adenocarcinoma	16	11	5	0.022
Squamous cell carcinoma	12	3	9	

**Figure 3:**
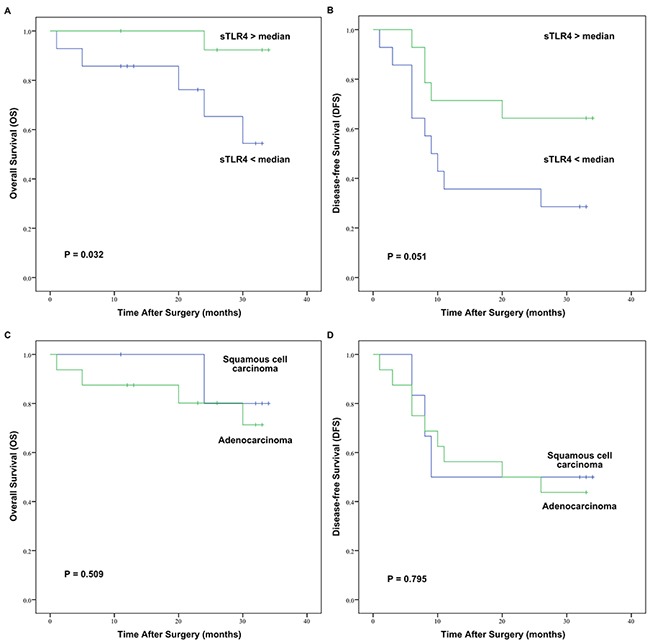
OS and DFS curves of 28 early-stage NSCLC patients after surgery assessed by Kaplan–Meier analysis according to serum sTLR4 levels or pathological types **A.** Patients with low serum levels of sTLR4 were significantly associated with poor OS (*P* = 0.032). **B.** Serum levels of sTLR4 were not significantly associated with DFS (*P* = 0.051). **C.** Pathological types were not significantly associated with OS (*P* = 0. 509). **D.** Pathological types were not significantly associated with DFS (*P* = 0.795).

Given the lack of clinically defined cutoff points for the serum levels of sTLR4 in NSCLC patients, the median expression levels of sTLR4 (33.8154 ng/mL for stage I and 48.9097 ng/mL for stage II) were used as cutoff points to define the sTLR4-low and sTLR4-high groups in early-stage NSCLC patients. The sTLR4-low group consisted of both stages I and II NSCLC patients with serum sTLR4 levels below the cutoff points. The sTLR4-high group consisted of both stages I and II NSCLC patients with serum sTLR4 levels above the cutoff points. The mean follow-up period was 26.0±10.1 months (range, 1.0–34.0 months). At the end of follow up, six deaths and 22 survivals were reported. The one-, two-, and three-year overall survival (OS) rates for patients of the sTLR4-high group were 100.0%, 100.0%, and 86.0%, respectively. For patients of the sTLR4-low group, the one-, two-, and three-year OS rates were 85.0%, 76.0%, and 42.0%, respectively. The mean OS of the sTLR4-high group was significantly longer than that of the sTLR4-low group (33.23±0.74 vs. 26.17±2.88 months, *P* = 0.032) (Figure 3A). The one-, two-, and three-year disease-free survival (DFS) rates for patients of the sTLR4-high group were 71.0%, 64.0%, and 64.0%, respectively, and 36.0%, 36.0%, and 24.0% for patients of the sTLR4-low group, respectively. Although the mean DFS in the sTLR4-high group (25.50±3.15 months) was longer than that in the sTLR4-low group (15.57±3.29 months), the difference was not significant (*P* = 0.051, Figure 3B).

As shown in Table 2, serum sTLR4 level was not associated with characteristics such as gender, age, and smoking status in early-stage NSCLC. Serum sTLR4 was significantly higher in early-stage squamous cell lung carcinoma patients than that in early-stage lung adenocarcinoma patients (*P* = 0.022). However, there was no significant difference in either OS (*P* = 0.509) or DFS (*P* = 0.795) between early-stage squamous cell lung carcinoma patients and early-stage lung adenocarcinoma patients (Figure 3C and 3D).

## DISCUSSION

The present study aimed to evaluate the clinical significance of serum sTLR4 in NSCLC. TLR4 has recently emerged as a key regulator of innate and adaptive immunity [16, 17]. Notably, the immune response is regulated at multiple levels, and the release of extracellular domains of immune receptors, including TLRs, represents one of the most important regulatory mechanisms [11, 15, 18–20].

In the present work, we evaluated serum levels of sTLR4 in 54 NSCLC patients and 13 healthy controls. Patients with NSCLC were characterized by significantly higher serum levels of sTLR4 in comparison with healthy controls. A positive correlation between serum sTLR4 and tumor stage was found in localized disease, whereas serum sTLR4 level declined significantly in patients with metastatic NSCLC. These results suggested that sTLR4 was involved in the carcinogenesis, progression, and metastasis of patients with NSCLC.

We also identified the prognostic role of serum sTLR4 in early-stage NSCLC patients who received surgical resection. Unfortunately, even surgical resection is a potentially curative therapy for early-stage NSCLC patients and has been confirmed to provide better survival outcomes; the postoperative recurrence rate remains higher in NSCLC patients than in patients with other types of cancer [21]. In the present study, we found that early-stage NSCLC patients with high serum sTLR4 at diagnosis had a superior OS after curative resection compared with those with low serum sTLR4.

The expression and activation of TLR4 have been reported on a variety of tumor and stromal cells in the tumor microenvironment [17, 22, 23]. The soluble form of TLR4 has been demonstrated to exert inhibitory activity on TLR signaling [11, 24–26]. One possible mechanism is that the complex formed by sTLR4 and MD-2 may block the interaction between membrane-bound TLR4 and its ligands [11]. For example, the sTLR4-MD-2 complex has been demonstrated to compete with wild-type TLR4-MD-2 receptor complex for LPS recognition [25].

HMGB1, a major endogenous ligand of TLR4, is one of the earliest identified and well-characterized alarmins [27–29]. HMGB1 expression is increased in the lungs of NSCLC patients and correlates with disease progression [29]. Serum levels of HMGB1 of patients with NSCLC have also been demonstrated to be significantly higher than those in healthy controls [12, 13]. However, contradictory data on the biomarker function of HMGB1 in NSCLC have been reported [12, 13]. The results of our present work demonstrated an increasing trend of serum HMGB1 from stage I to stage III in patients with NSCLC without statistical significance. Serum HMGB1 level was significantly higher only in stage IV than in any other stage.

In addition to HMGB1, sTLR4 also has other endogenous ligands, including several heat shock proteins (HSPs) and S100 proteins. For example, HSP27 is reportedly involved in cancer metastasis and prognosis, and the serum levels of HSP27 have been demonstrated to be significantly increased in cancer patients [30]. As another example, the S100A8/S100A9 complex has also been identified as a potent amplifier of inflammation in tumorigenesis [31].

These endogenous ligands of TLR4 bind and activate TLR4 on tumor and stromal cells during cancer development and contribute to increased evasion of immune surveillance [17, 22, 23]. Therefore, increasing levels of serum sTLR4, by blocking the binding of TLR4 ligands to membrane-bound TLR4, may inhibit intracellular signaling through membrane-bound TLR4 and then dampen the pro-inflammatory tumor microenvironment. The results of the present study indicated that serum sTLR4 played an active role in NSCLC pathogenesis and could become a serum biomarker for NSCLC to evaluate disease progression and predict the outcomes for early-stage patients who received surgical resection. More work needs to be conducted to decipher the exact mechanisms by which sTLR4 regulate tumor immunity.

A possible shortcoming of our study was that the levels of sTLR4 or HMGB1 may be affected by many factors, such as infection, certain medicines, or even obesity [32]; hence, large variations in serum levels of sTLR4 and HMGB1 were found between NSCLC patients even within the same stages. These variations may potentially dilute the value of using serum sTLR4 as a prognostic marker. However, after application of the exclusion criteria of this study, we clearly demonstrated that serum levels of sTLR4 were positively correlated with tumor stages in NSCLC patients with local disease. For patients with metastatic NSCLC, the serum sTLR4 levels declined significantly, whereas the serum HMGB1 levels rose. Most importantly, our study also suggested that serum sTLR4 may be useful for evaluating disease progression and predicting the outcomes for early-stage patients undergoing surgical resection.

The present study only evaluated the clinical significance of serum sTLR4 and HMGB1 in a relatively small group, so further studies are required to validate these results in large-scale and compare them with other existing biomarkers of NSCLC, such as squamous cell carcinoma antigen, cytokeratin 19 fragment 21–1, carcinoembryonic antigen, cancer antigen-125, or carbohydrate antibody 19-9 [33].

Taken together, our present work, as well as our previous work on HMGN1 [14], suggested that the alarmin system, which consists of alarmins and their soluble or membrane-bound receptors, hold great potential as ideal biomarkers to predict prognosis, monitor disease progression, or even access therapeutic effects of NSCLC patients.

## MATERIALS AND METHODS

### Patients

A total of 54 NSCLC patients and 13 healthy volunteers were enrolled in the Department of Biotherapy, Tianjin Medical University Cancer Institute and Hospital (TMUCIH), Tianjin, China, between January 2012 and December 2013. All patients were diagnosed for the first time during the enrollment period and classified into TNM stages. The following patients were excluded from this study: patients with previous or simultaneous cancers; patients who suffered from concomitant diseases that can influence the levels of sTLR4 or HMGB1, such as trauma/fracture, inflammatory systemic disease, or infection; and patients who were treated with medication that can influence the levels of sTLR4 or HMGB1 within one month. Postoperative treatment for patients with stage I or II NSCLC was strictly based on the National Comprehensive Cancer Network Clinical Practice Guideline in NSCLC. The study was approved by the ethics committee of TMUCIH according to the principles expressed in the Declaration of Helsinki. All the patients and healthy volunteers provided informed consent prior to participation.

### Samples collection and preparation

All blood samples were obtained prior to the initiation of any treatment and collected in non-heparinized tubes. The clinical and demographic features of patients with different stages of NSCLC or healthy volunteers were recorded during blood collection. Serum samples were prepared by centrifugation for 15 min at 1000 ×g, divided into four to five aliquots, and stored at −80°C until assayed.

### Enzyme-linked immunosorbent assay (ELISA)

The concentrations of sTLR4 were measured by ELISA (KA1238, Abnova) according to the manufacturer's instructions. In brief, 100 μL of standard, blank, or sample was added to an appropriate well of a microtiter plate that was pre-coated with an antibody specific to TLR4. The plate was sealed and incubated at 37°C for 2 h. The liquid of each well was removed, and 100 μL of biotin-conjugated polyclonal antibody specific for TLR4 was added to each well. The plate was sealed again and incubated at 37°C for 1 h. After three washes, 100 μL of horseradish peroxidase-conjugated avidin was added to each well. The sealed plate was incubated at 37°C for another hour. After five washes, 90 μL of TMB substrate solution was added to each well. The plate was then covered and incubated about 15–30 min at 37°C and protected from light before adding 50 μL of sulfuric acid stop solution to each well. The optical density of each well was read at 450 nm with a microplate reader (Synergy HT, BioTek, Winooski, VT, USA). The concentration of sTLR4 in the serum samples was then calculated according to the standard curve.

The serum levels of HMGB1 were quantified by sandwich ELISA (HMGB1 ELISA Kit II, Shino-Test Corporation, Japan) in a modified procedure suggested by the manufacturer. In brief, 10 μL of sample was added to an appropriate well of a microtiter plate to immobilize anti-HMGB1 antibody on the well together with 100 μL of sample diluents. Subsequently, the plate was sealed and incubated for 24 h at 37°C to allow HMGB1 to specifically bind to the antibody. After five washes, 100 μL of peroxidase-conjugate secondary antibody was added to each well. The plate was then sealed and incubated at 25°C for 2 h. After another five washes, 100 μL of substrate solution was added to each well, and the plate was incubated for 30 min at room temperature. The optical density of each well was read at 450 nm after adding 100 μL of stop solution to each well.

### Follow up

All patients visited TMUCIH for follow up every three months in the first two years, and every six months in three to five years after curative pneumonectomy. Follow-up evaluations included medical interviews, physical examination, routine laboratory testing, and chest radiography. The last follow-up date for patients still alive was March 2015. Causes of death and sites of recurrence were confirmed by hospital records, death certificates, and radiological findings. OS reflected the interval between the time of pneumonectomy and time of death or the last date of follow up. DFS was the time of pneumonectomy to the time when recurrence was diagnosed or to the last date of follow up. Recurrent tumors were treated at the Department of Biotherapy, TMUCIH.

### Statistical analysis

All statistical analysis was performed with the Statistical Package for the Social Sciences (SPSS) version 13.0 (Chicago, IL, USA). Numerical data were expressed as the mean ± standard deviation. The Kolmogorov–Smirnov test was applied to evaluate normality of data. Categorical variables were compared by chi-square test or Fisher's Exact Test. Comparisons of numerical data were performed by independent sample t-test, Wilcoxon Rank Sum Test, or one-way ANOVA with Student Newman–Keuls test for pairwise comparison. The correlations between sTLR4 and other variables were measured by Pearson correlation analysis or Spearman correlation analysis. OS and DFS were calculated by the Kaplan–Meier method and compared by log-rank test. A two-tailed *P* value less than 0.05 was considered statistically significant.
